# Liver resection versus transarterial chemoembolisation for the treatment of intermediate hepatocellular carcinoma: a systematic review and meta-analysis

**DOI:** 10.1097/JS9.0000000000000344

**Published:** 2023-04-14

**Authors:** Aleksandar Bogdanovic, Jelena Djokic Kovac, Predrag Zdujic, Uros Djindjic, Vladimir Dugalic

**Affiliations:** a Clinic for Digestive Surgery; b Center for Radiology and Magnetic Resonance Imaging, University Clinical Center of Serbia; c School of Medicine, University of Belgrade, Belgrade, Serbia

**Keywords:** Barcelona Clinic Liver Cancer staging, hepatocellular carcinoma, intermediate-stage B, liver resection, transarterial chemoembolization

## Abstract

Transarterial chemoembolisation (TACE) is the primary treatment for intermediate-stage hepatocellular carcinoma (HCC), according to the updated Barcelona Clinic Liver Cancer (BCLC) staging system. Although growing evidence favours liver resection (LR) over TACE for intermediate-stage HCC, the best treatment option remains controversial. This meta-analysis aimed to compare the overall survival (OS) after LR versus TACE for intermediate-stage HCC.

**Methods:**

A comprehensive literature review of PubMed, Embase, Cochrane Library, and Web of Science was performed. Studies that compared the efficacy of LR and TACE in patients with intermediate (BCLC stage B) HCC were selected. According to the recent updated BCLC classification, intermediate stage of HCC was defined as follows: (a) four or more HCC nodules of any size, or (b) two or three nodules, but if at least one tumour is larger than 3 cm. The main outcome was OS, expressed as the hazard ratio.

**Results:**

Nine eligible studies of 3355 patients were included in the review. The OS of patients who underwent LR was significantly longer than that of patients who underwent TACE (hazard ratio=0.52; 95% CI: 0.39–0.69; I^2^=79%). Prolonged survival following LR was confirmed after sensitivity analysis of five studies using propensity score matching (HR=0.45; 95% CI: 0.34–0.59; *I* ^2^=55%).

**Conclusion:**

Patients with intermediate-stage HCC who underwent LR had a longer OS that those who underwent TACE. The role of LR in patients with BCLC stage B should be clarified in future randomised controlled trials.

## Introduction

Highlights

- Transarterial chemoembolisation is the primary treatment option for intermediate-stage hepatocellular carcinoma (HCC) according to the updated Barcelona Clinic Liver Cancer staging system, although recent evidence favours liver resection (LR) in terms of long-term survival.
- Overall survival of patients with intermediate-stage HCC who underwent LR is longer than in those who underwent transarterial chemoembolisation.
- LR should play an important role in the treatment strategy for intermediate-stage HCC.
- The datasets generated or analysed during the current study are available from the corresponding author on reasonable request.

Hepatocellular carcinoma (HCC) is the third leading cause of cancer-related deaths globally^1^. In 2020, HCC was responsible for 830 180 deaths, and 905 677 new cases worldwide^2^. Despite epidemiological progression during the last decades, HCC incidence is still growing, and more than 1.3 million cases are expected by 2040^3^. HCC is characterised by aggressive nature, rapid progression, and poor overall median survival of up to 2 years^4^. Without strict surveillance protocols, initial curative treatment is possible in only 10–30% of patients with HCC in Western countries^5^.

The Barcelona Clinic Liver Cancer (BCLC) classification based on general status, liver function, and tumour burden has been endorsed by the European Association for the Study of Liver and the American Association for the Study of Liver Disease (AASLD) as the primary staging system, prognostic classifier and treatment algorithm for HCC^6^. According to the updated EASL clinical practice guidelines, liver resection (LR) is recommended only for early-stage tumours (BCLC 0-A), and transarterial chemoembolisation (TACE) for multinodular HCC (intermediate B stage)^7^. In the latest AASLD guidelines, TACE should be considered for patients with intermediate-stage HCC who are not eligible for curative treatments^8^. Additionally, TACE is the first treatment option for multinodular HCC according to the Japanese Consensus-Based Clinical Practice Guidelines; however, resection is sometimes performed even when more than four nodules are present^9^.

Some meta-analyses have considered solitary large HCC as intermediate-stage disease^10^,^11^. However, solitary HCC, regardless of size, was recently classified as early-stage disease. Therefore, it should be treated with potentially curative resection in selected cases^7^. However, the intermediate B stage still remains a heterogeneous group of patients in terms of the number and size of nodules, Child-Pugh score, and general condition. Several single reports have shown survival benefits in patients with multinodular HCC who were managed by curative-intent liver surgery compared with by TACE^12^,^13^. Nevertheless, the improved survival after LR is more evident in earlier substages of intermediate HCC^14^. Therefore, there is no sufficient evidence in favour of LR compared with TACE for intermediate-stage HCC.

This study aimed to compare the overall survival (OS) between patients treated with LR or TACE for intermediate-stage HCC.

## Method

### Systematic review of literature

A comprehensive systematic literature review of the PubMed, Embase, Cochrane Library, and Web of Science databases was conducted to identify studies comparing the efficacy of LR and TACE in patients with intermediate-stage HCC. Studies published until 12 December 2021 were considered.

A literature search was performed using the following keywords and strategies: (liver cancer OR hepatocellular carcinoma OR HCC) AND (intermediate OR BCLC B) AND (transarterial chemoembolisation OR TACE) AND (hepatectomy OR hepatic resection OR LR OR liver surgery).

The work has been reported in line with PRISMA (Preferred Reporting Items for Systematic Reviews and Meta-Analyses), Supplemental Digital Content 1, http://links.lww.com/JS9/A333 and AMSTAR (Assessing the methodological quality of systematic reviews) Guidelines^15^,^16^. Supplemental Digital Content 2, http://links.lww.com/JS9/A334 The study review protocol was registered at Research Registry (unique identifying number: reviewregistry1488).

### Study selection

The inclusion criteria were as follows: (1) population: patients diagnosed with BCLC stage B HCC (intermediate B stage of HCC was defined based on the updated BCLC classification according to one of two criteria: (a) two or three nodules, but if at least one tumour is larger than 3 cm, and (b) four or more HCC nodules of any size); (2) intervention: LR; (3) comparison: TACE alone; and (4) outcome: OS after the interventions expressed as hazard ratio (HR).

Exclusion criteria were as follows: (1) studies reporting patients without HCC or with recurrent HCC; (2) non-comparative studies including abstracts, conference articles, opinions, case series, reviews, and meta-analyses; (3) studies reporting LR combined with preoperative or postoperative TACE; (4) studies comparing multimodal treatment other than TACE; (5) studies without BCLC classification; (6) studies without valid survival data; and (7) studies lacking key data for HR extraction.

All publications were screened for eligibility in two phases by two independent reviewers (B.A. and Z.P.) according to the inclusion and exclusion criteria. Disagreements in each step were resolved by discussion between the two independent reviewers, with the opinion of a third reviewer (D.V.), or by consensus.

### Data extraction and quality assessment

The following variables were extracted from full-text publications: characteristics of the included studies (year of study, design, state, study period, number of patients in the LR and TACE groups), and patients’ baseline clinical characteristics for LR and TACE before and after propensity matching [age, sex, hepatitis B virus (HBV), hepatitis C virus (HCV), tumour number and diameter, survival outcomes for both groups before and after propensity matching (median follow-up and 1-year, 3-year, and 5-year survival)]. The survival outcome data, including HR with a corresponding 95% CI, were also extracted from related articles. We used the modified Newcastle-Ottawa score to estimate the bias risk in non-randomised controlled trials (Supplemental Table S1, Supplemental Digital Content 3, http://links.lww.com/JS9/A335)^17^.

### Statistical analysis

The main outcome was OS, which was expressed as log HR and standard error. For publications without HR and 95% CI reported, data were obtained by using an online application which was developed for data extraction from systematic review graphs^18^. Thereafter, the log HR and standard error were calculated using a calculator formulated by Tierney *et al*.^19^. Review Manager (version 5.3 software, Cochrane Collaboration) was used to calculate the summary HR effect size, and heterogeneity was assessed using the Cochran Q test and I^2^ statistic. Heterogeneity was defined as I^2^ greater than 50%^20^ or *P* value less than 0.10. Owing to the presence of heterogeneity, a random-effects model was used. Two separate forest plots were constructed for the data before and after propensity matching with HR (box), 95% CI (lines), and weight (box size) for each trial. Diamonds represent the overall effect size. Publication bias was assessed by using funnel plot asymmetry. Statistical significance was set at *P* less than 0.05.

## Results

### Systematic review

**Figure 1:**
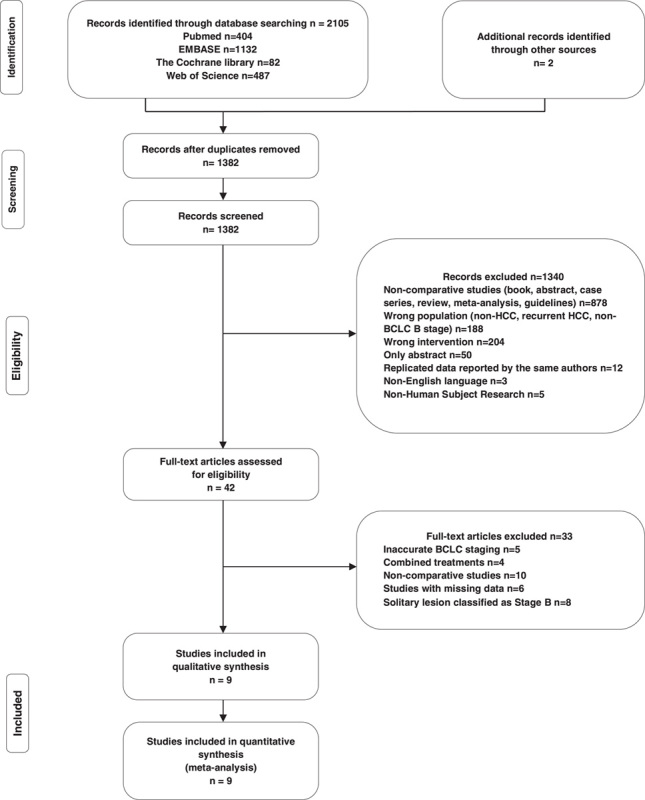
Flow chart. BCLC, Barcelona Clinic Liver Cancer; HCC, hepatocellular carcinoma.

In total, 2107 potentially eligible articles were identified. After exclusion of duplicates, 1382 titles and abstracts were screened. After reading the titles and abstracts, 1340 articles were excluded as they were non-comparative studies, examined the wrong population (patients without HCC, recurrent HCC, non-BCLC B stage), compared interventions other than LR or TACE, or were published only as abstracts. Of the remaining full-text articles, 33 were excluded due to inaccurate BCLC stages, use of combined treatments, or solitary lesions classified as stage B (excluded full-text articles are summarised in Supplemental Table S2, Supplemental Digital Content 4, http://links.lww.com/JS9/A336). A total of nine articles were finally included in the systematic review and meta-analysis^12^,^13^,^21^–^27^. The flow chart representing the study selection steps is shown in Fig. 1.

The characteristics of the nine publications are presented in detail in Table 1. The studies were published between 2011 and 2021 and included 3355 patients, with a minimum sample size of 80 and a maximum of 942. Of these, 1130 patients were treated with LR and 2225 with TACE. All studies were retrospectively designed, except for one prospective study and one randomised control trial. Most studies were single-centre studies, while two were multicentric, and one was double-centre. Most studies had been conducted in China^21^–^23^,^26^,^27^.

**Table 1:** Characteristics of included studies.

First Author	Year	Design	Centre	State/region	Study period	No. patients	LR group	TACE group
Ruben Ciria^12^	2015	Retrospective	Single	Spain	Jan 2007–Dec 2012	80	36	44
Jun Young Kim^13^	2016	Retrospective	Multi	South Korea	Jan 2005–Dec 2009	277	52	225
Linbin Lu^21^	2021	Retrospective	Single	China	Jan 2007–May 2012	942	225	717
Jun Luo^22^	2011	Prospective	Single	China	Jan 2004–Dec 2006	168	85	83
Yufu Peng^23^	2019	Retrospective	Single	China	Apr 2015–Oct 2018	224	75	149
Toshifumi Tada^24^	2017	Retrospective	Multi	Japan	2000–2015	489	170	319
Chih-Wen Lin^25^	2020	Retrospective	Single	Taiwan	2011–2018	371	140	231
Wei Xu^26^	2018	Retrospective	Double	China	Jan 2000–Jan 2012	631	259	372
Lei Yin^27^	2014	RCT	Single	China	Nov 2008–Sep 2010	173	88	85

LR, liver resection; RCT, randomized controlled trial; TACE, transarterial chemoembolisation.

### Baseline clinical characteristics and survival outcomes before matching

Most patients were aged 51–60 years. The proportion of male patients varied from 75 to 95%. Most patients in the LR and TACE groups had HBV infection (13.9–99%)^21^, while chronic HCV infection incidence varied (1.1%^27^–43.2%^12^). The percentage of patients with four or more tumours was higher in the TACE group than in the LR group. The maximum tumour size was similar in both the groups. The patient baseline characteristics are presented in Table 2.

**Table 2:** Baseline patients’ clinical characteristics.

	Age (years)		Male (%)		HBV (%)		HCV (%)		Tumour number		Maximal tumour diametre	
First Author	LR	TACE	LR	TACE	LR	TACE	LR	TACE	LR	TACE	LR	TACE
Ruben Ciria^12^	67±10	65±9	77.8	75	13.9	15.9	27.8	43.2	1.5±1^c^	2±1^c^	nr	nr
Jun Young Kim^13^	54.6±8.6	58.3±10	90.4	85.8	nr	nr	nr	nr	1.9	24	50^b^	45.3^b^
Linbin Lu^21^	50.9±12.6	53.9±12.3	90,7	91.2	99	92	nr	nr	36.9	66.9	6.4±2.8	7.5±3.8
Jun Luo^22^	47.5±12.8	50.9±11.2	82.3	95.2	82.3	91.6	2.3	4.8	35.3	48.2	8.7±3.5	7.8±2.5
Yufu Peng^23^	55 (31–76)^a^	55 (29–79)^a^	80	87	75	75	nr	nr	25	23	5 (3–15,5)^a^	6 (2–20)^a^
Toshifumi Tada^24^	nr	nr	nr	nr	nr	nr	nr	nr	nr	nr	nr	nr
Chih-Wen Lin^25^	62 (35–82)^a^	64 (29–91)^a^	83.6	74.9	50	44.6	21.4	38.9	35^d^	77.1^d^	8.2±3.3	7±3.8
Wei Xu^26^	56.7±12.4	55.6±11.1	83	85	68	72	7	9	1.9	10.2	8.3±3.4	8.6±3.5
Lei Yin^27^	51.6±9	54±9.5	93.1	92.9	92	90.6	3.4	1.1	12	9.4	7.3±2.5	7.4±2.3

HBV, hepatitis B virus; HCV, hepatitis C virus; LR, liver resection; nr, not reported; TACE, transarterial chemoembolisation.

Values of age and maximal tumour diametre are expressed as mean±SD unless indicated otherwise;

^a^Median (range).

b Percentage of patients with maximal tumour diametre ≥5 cm.

Values of tumour number are expressed as percentage of patients with four or more tumours unless indicated otherwise;

c Mean±SD.

d Percentage of patients with three or more tumours.

The median follow-up was reported for five studies and varied from 13 to 37.6 months^12^,^13^,^23^,^24^,^26^. One-year, 3-year, and 5-year survival rates were reported in seven studies and were higher in the LR group than in the TACE group. Survival outcomes are shown in Table 3. Publication bias was not significant according to funnel plot diagram (Supplemental Figure S2, Supplemental Digital Content 5, http://links.lww.com/JS9/A337).

**Table 3:** Survival outcome.

	Median follow-up (months)			1-year survival (%)		3-year survival (%)		5-year survival (%)		
First Author	Overall	LR	TACE	LR	TACE	LR	TACE	LR	TACE	*P*
Ruben Ciria^12^	28.2	nr	nr	83.3	68.2	52.8	47.7	44.4	38.6	0.229
Jun Young Kim^13^	30.0	nr	nr	92.3	78.2	65	39.2	51.8	27.9	0.002
Linbin Lu^21^	nr	67.4	18.5	85.8	76.9	68.6	52.7	63.3	46.7	<0.0001
Jun Luo^22^	nr	nr	nr	70.6	67.2	35.3	26	23.9	18.9	0.26
Yufu Peng^23^	13	nr	nr	nr	nr	nr	nr	nr	nr	nr
Toshifumi Tada^24^	26	nr	nr	nr	nr	nr	nr	nr	nr	nr
Chih-Wen Lin^25^	nr	39.0	22	89.2	69.5	69.4	37.0	61.2	15.2	<0.0001
Wei Xu^26^	37.6	nr	nr	86.5	73.9	53.8	45.7	33.8	28.9	0.03
Lei Yin^27^	nr	33.3	13.5	76.1	51.8	63.5	34.8	51.5	18.1	<0.001

LR, liver resection; nr, not reported; TACE, transarterial chemoembolisation.

### Baseline clinical characteristics and survival outcome after matching

Matching of the patients’ baseline clinical characteristics was performed in five studies (Supplemental Table S3, Supplemental Digital Content 6, http://links.lww.com/JS9/A338). Most patients were 51–60 years old; Tada *et al*.^24^ reported older patients in both groups. After matching, the frequency of male patients included in studies within the groups varied from 77%^25^ to 95%^21^. Chronic HBV infection was present in 14.4%^24^–98.7%^21^ patients. Only two studies reported chronic HCV infection distribution after matching^24^,^25^. The percentage of patients with four or more tumours and tumour diameters were similar in both groups after matching.

The survival outcomes after matching are listed in Supplemental Table S4, Supplemental Digital Content 7, http://links.lww.com/JS9/A339. Two studies reported longer 3-year and 5-year survival rates in the LR group than those in the TACE group. Publication bias was not significant according to funnel plot diagram (Supplemental Figure S3, Supplemental Digital Content 8, http://links.lww.com/JS9/A340).

### Intervention-associated OS in Patients with Intermediate-Stage (BCLC B) HCC

**Figure 2:**
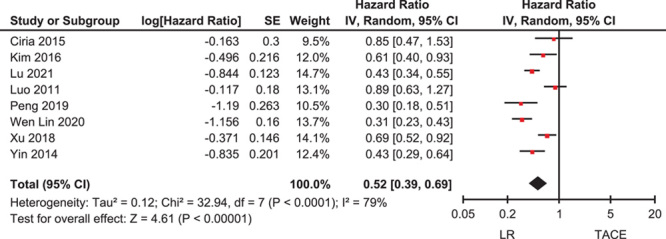
Overall survival according to therapeutic intervention (LR versus TACE) in patients with intermediate-stage hepatocellular carcinoma. LR, liver resection; TACE, transarterial chemoembolization.

A meta-analysis was performed to examine the OS according to intervention (LR versus TACE) in patients with intermediate-stage (BCLC B) HCC. A total of eight studies had OS as an outcome before propensity matching^12^,^13^,^21^–^23^,^25^–^27^. OS was longer in patients who underwent LR than in those who underwent TACE (HR=0.52, 95% CI: 0.39–0.69) (Fig. 2). There was a high degree of heterogeneity in the OS analysis (*I* ^2^=79%).

The sensitivity analysis, including one study with HR after propensity matching, showed a similar result (HR=0.52, 95% CI:0.41–0.67) (Supplemental Figure S1, Supplemental Digital Content 9, http://links.lww.com/JS9/A341)^24^.

### Intervention-associated OS after propensity matching in patients with intermediate-stage (BCLC B) HCC

**Figure 3:**
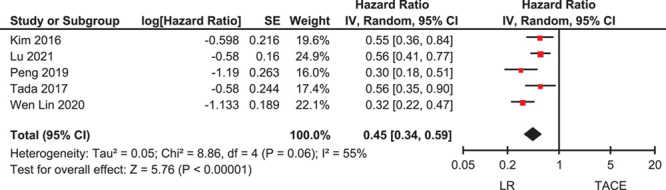
Overall survival according to therapeutic intervention (LR versus TACE) after propensity matching in patients with intermediate-stage hepatocellular carcinoma. LR, liver resection; TACE, transarterial chemoembolization.

A meta-analysis was performed to examine the OS according to intervention (LR versus TACE) after propensity matching in patients with intermediate-stage (BCLC B) HCC. Four studies had OS as an outcome after propensity matching^21^,^23^–^25^, while one study reported HR related to treatment modality after a matched analysis (matched by age, Child-Pugh score, AFP, tumour size, tumour number, underlying liver disease, PT-INR and interactions between tumour number and INR ratio)^13^. OS was longer in patients who underwent LR than in those who underwent TACE (HR=0.45, 95% CI: 0.34–0.59) (Fig. 3). There was a high degree of heterogeneity in the OS analysis (*I* ^2^=55%).

## Discussion

The present meta-analysis showed longer OS of patients with intermediate-stage HCC following LR compared to TACE, with mortality risk reduced to almost half (HR=0.52); significantly better survival was demonstrated in five out of the seven studies. However, Ciria and colleagues and Luo and colleagues did not confirm the superiority of LR over TACE in terms of long-term survival^12^,^22^. To reduce the confounding effect of pretreatment patient characteristics, propensity score matching was performed in five studies. The sensitivity analysis established the survival benefits of patients undergoing LR.

Four electronic databases were systematically searched for full-text eligible studies that included OS rates and summarised HR, in contrast to most historical meta-analyses which have used only odds ratio without accounting for time in a forest plot.

Solitary large HCC exceeding 5 cm were previously classified as BCLC stage B. Although large-sized HCC are more commonly associated with macrovascular invasion and a higher probability of intrahepatic and extrahepatic micrometastatic disease, significantly better long-term survival is seen after curative-intent hepatectomy in contrast to after TACE^28^. Prolonged OS after LR over TACE has been confirmed by a recent meta-analysis which considered solitary HCC nodules as intermediate-stage disease^10^,^11^,^29^. Nevertheless, long-term outcome results favouring LR over TACE were questioned by confounding misclassification. In 2018, EASL recommended classification of solitary HCC, regardless of tumour size, as early-stage A^7^. To overcome the over-staging bias, we conducted a systematic review and subsequent meta-analysis of studies using strict selection criteria, and excluded a large subset of patients with single large HCC. In a recent meta-analysis by Labgaa *et al*.^30^, similar strict criteria were defined. However, an additional five studies were included in the current analysis with a total of 2336 patients; four of these were published after, while one prospective study was published before the meta-analysis by Labgaa *et al*.^21^–^23^,^25^,^26^.

Intermediate-stage HCC is characterised by excessive heterogeneity, including patients from near-early to near-advanced stages. Tumour burden, defined by the number and size of nodules, varies considerably within stage B. Liver function can also differ if it is scored as Child-Pugh A or B. Therefore, the subset of patients with different prognoses needs an appropriate therapeutic approach. Traditionally, only TACE has been established as the first-line treatment for this heterogeneous group of patients after the first randomised controlled trial by Llovet *et al*.^31^. However, treatment deviations from the BCLC recommendations exceed 50% of cases in referral centres worldwide^32^. Although the BCLC classification has been updated several times since its origin, the treatment algorithm for patients with intermediate-stage HCC remains controversial. Choosing the appropriate treatment is a complex process requiring the integration of basic parameters, such as tumour burden, liver function, and general condition, with the individual medical profile of each patient, to provide a personalised approach^33^. Therefore, various subclassifications have been proposed to overcome difficulties related to patient prognostication and treatment allocation in the clinical setting^34^–^36^. Bolondi *et al*.^34^ proposed subclassification of intermediate-stage HCC into 4 categories B1-4. For B1 substage (Child-Pugh score 5, 6, or 7; tumour load within up-to-seven criteria), TACE was recommended as the first-line treatment, while if patients are beyond up-to-seven rule (B2, B3, and B4) TACE or best supportive care are recommended. More recent study by Zhaohui *et al*.^37^, showed that HCC patients at B1 stage were benefited from hepatic resection and had similar survival to BCLC-A stage patients. More recently, the Kinki criteria have been proposed by Kudo *et al*.^36^, which suggests curative-intent treatment for B1 patients with Child-Pugh score 5–7 and tumour burden ‘beyond Milan’ and within ‘up-to-seven’ criteria. LR, ablation, and super-selective TACE were designated as curative modalities and included in the Kinki staging system as first-line therapies. We can clearly conclude that LR is superior over TACE for B1 substage HCC that is characterised by Child-Pugh score 5–7 and tumour load within up-to-seven criteria. In contrast, the latest BCLC 2022 update does not offer LR as a potentially curative treatment to patients with BCLC B stage HCC. According to the BCLC update, the intermediate-stage is divided into three subgroups and have the following treatment recommendations: liver transplantation, TACE, and systemic therapy^6^.

Debate regarding the optimal treatment approach for the more advanced stages B and C has long been initiated. In 2013, Torzilli *et al*.^38^ conducted a multicentric retrospective analysis of nine tertiary referral centres worldwide to provide real-world information on surgical practices for HCC, challenge the BCLC strategy, and eventually request an update of treatment recommendations. The authors demonstrated post-surgical 5-year survival of 57% and 38% in patients with BCLC stages B and C, respectively, who were considered unsuitable for hepatectomy according to EASL guidelines; the 3-year expected survival had been 10–40%. After 5 years, 38% of the patients with advanced-stage disease were alive, and 53% of them were alive if cirrhosis was absent. However, the results of this study were disputed by Bruix and Fuster as too robust for interpretation and conclusions^39^. A major drawback of this study was that a large proportion of patient with HCC stages B and C with impaired liver function or technical unresectability were excluded from the analysis. HCC was staged based on specimen analysis; therefore, pretreatment classification and imaging-based staging strategies at the time of treatment initiation were mismatched.

In a study by Ciria *et al*.^12^ long-term survival following LR for intermediate-stage HCC was not superior to that of TACE. Nevertheless, patients with B1 intermediate-stage HCC undergoing LR or TACE had a satisfactory 5-year survival rate of 63.2% and 62.5%, respectively. Therefore, LR may be considered for substage B1 HCC. LR did not yield a survival advantage over TACE for intermediate-stage HCC in the only prospective non-randomised analysis, but aggressive hepatic resection should be recommended for patients with a good response to TACE^22^.

There is a huge discrepancy in liver surgery for intermediate HCC between the recommended therapeutic algorithm and current practice worldwide. Thus, Glantzounis *et al*.^40^ conducted a systematic review of LR’s role in patients with intermediate-stage B HCC and concluded that long-term survival after LR was much better than after the recommended strategy. Yang *et al*.^41^ provided a similar conclusion; however, LR is only advised for patients with well-preserved liver function. If BCLC treatment recommendations were strictly enforced, many patients suitable for hepatectomy would be excluded with no chance of prolonged survival. However, LR for HCC is still associated with higher perioperative morbidity and mortality due to the underlying liver disease. Regardless of refinements in liver surgery and perioperative intensive care, the morbidity rate is still relatively high, ranging between 5% and 41%; the mortality is nearly 3% in referral centres^42^,^43^. Nonetheless, several TACE-related adverse events or mortality should also be considered when deciding the treatment approach. High recurrence rates and low disease-free survival were also seen in patients with stage B HCC who underwent LR. Therefore, rigorous postoperative follow-up is recommended; if early recurrence is detected, additional treatment, including hepatic intra-arterial therapy, tumour ablation, or systemic therapy will be needed. Moreover, TACE is not an alternative for LR; the methods may be combined consecutively to prolong OS and improve patients’ quality of life.

Currently, data from prospective randomised studies are lacking, and the results of retrospective observational studies should be interpreted with caution. Furthermore, as large discrepancies exist in the recommended treatments, the decision to perform liver surgery must be individualised, tailoring the approach to specific patient needs. The perioperative risk of morbidity and mortality must not be neglected, as well as the high risk of early recurrence after surgery. Hence, the question of identifying patients who could benefit from the surgical approach remains. Undoubtedly, LR should play an important role in the treatment strategy for intermediate HCC; however, careful patient selection remains the key in providing a better prognosis and quality of life. LR should be proposed as an optimal treatment model in the future for stage B HCC based on evidence from randomised controlled trials and validated, refined, subclassification of the BCLC B stage. Identification of molecular biomarkers and knowledge of the cancer microenvironment will clarify the LR’s importance in the management of intermediate-stage HCC through precision medicine.

The present study has several limitations. Most of the included studies had a retrospective design. Selection bias is a major drawback, as patients do not have an equal chance of receiving each treatment model. Furthermore, patients fulfilling the criteria for TACE may not be optimal candidates for surgery; conversely, patients selected for surgery may have contraindications for TACE. To reduce the treatment assignment bias, we performed a sensitivity analysis of the studies that conducted propensity score matching. Second, there was a high degree of heterogeneity between the studies. The study patients were a heterogeneous subgroup classified as having intermediate-stage disease. Furthermore, the number of patients from stage B who were not considered for LR or TACE were unknown. This excluded population could have had a significant impact on the study results. Third, most studies originated in the East, where a high prevalence of chronic HBV infection exists. In contrast, Western countries have characteristic underlying aetiologies of HCV infection and non-alcoholic fatty liver disease. The presence of liver cirrhosis was also reported using descriptive statistics, but the confounding effects of these factors were not analysed.

## Conclusion

The present systematic review and meta-analysis showed a longer OS for patients with intermediate-stage HCC who underwent LR than for those who underwent TACE. There is a great need for randomised controlled studies and new biomarkers to better stratify this subgroup of patients, clarify LR’s roles in BCLC stage B HCC, and refine the selection criteria for patients who could have improved survival after surgery.

## Ethical approval

This study is a meta-analysis and ethical approval is not applicable.

## Source of funding

No funding.

## Author contribution

A.B.: conceptualization, formal analysis, methodology, writing—original draft. P.Z.: data curation, formal analysis, methodology, writing—original draft. U.D.: data curation, formal analysis, methodology. J.K.D.: methodology, validation, writing—review and editing. V.D.: formal analysis, validation, writing—review and editing.

## Conflicts of interest disclosure

The authors have no conflicts of interest to disclose.

## Research registration unique identifying number (UIN)

1. Name of the registry: Research Registry.
2. Unique Identifying number or registration ID: reviewregistry 1488.
3. Hyperlink to specific registration (must be publicly accessible and will be checked): https://www.researchregistry.com/browse-theregistry#registryofsystematicreviewsmetaanalyses/registryofsystematicreviewsmetaanalysesdetails/638a3c3f6bcc400021edf985/.

## Guarantor

Aleksandar Bogdanovic.

## Provenance and peer review

Not commissioned, externally peer-reviewed.

## Supplementary Material
