# Supplementary material for: Liver resection versus transarterial chemoembolisation for the treatment of intermediate hepatocellular carcinoma: a systematic review and meta-analysis
Source: Int J Surg. 2023 Apr 14;109(5):1439–46. doi: 10.1097/JS9.0000000000000344 (PMC10389385; doi:10.1097/JS9.0000000000000344)
Supplement: Supplementary file 3 [file js9-109-1439-s003.docx]

Supplemental Table S1. Modified Newcastle-Ottawa Quality Assessment Scale for included non-randomized studies

| First Author | Selection | | | | Comparability | Outcome | | |  |
| --- | --- | --- | --- | --- | --- | --- | --- | --- | --- |
|  | Representativeness of the Exposed Cohort | Selection of the Non-Exposed Cohort | Ascertainment of Exposure | Outcome of Interest Was Not Present at Start of Study |  | Assessment of Outcome | Follow-Up Duration | Adequacy of follow-up | Overall score |
| Ruben Ciria^12^ | * | * | * | * | - | * | * | - | 6 |
| Jun Young Kim^13^ | * | * | * | * | * | * | * | - | 7 |
| Linbin Lu^21^ | * | * | * | * | ** | * | * | - | 8 |
| Jun Luo^22^ | * | * | * | * | - | * | * | * | 7 |
| Yufu Peng^23^ | * | * | * | * | ** | * | * | * | 9 |
| Toshifumi Tada^24^ | * | * | * | * | ** | * | * | - | 8 |
| Chih-Wen Lin^25^ | * | * | * | * | ** | * | - | - | 7 |
| Wei Xu^26^ | * | * | * | * | - | * | * | - | 6 |
