# Supplementary material for: Liver resection versus transarterial chemoembolisation for the treatment of intermediate hepatocellular carcinoma: a systematic review and meta-analysis
Source: Int J Surg. 2023 Apr 14;109(5):1439–46. doi: 10.1097/JS9.0000000000000344 (PMC10389385; doi:10.1097/JS9.0000000000000344)
Supplement: Supplementary file 4 [file js9-109-1439-s004.docx]

Supplemental Table S2. Excluded full-text articles

| First Author | Title | Reason for exclusion |
| --- | --- | --- |
| Adhoute X | Barcelona clinic liver cancer nomogram and others staging/scoring systems in a French hepatocellular carcinoma cohort | Study with missing data |
| Aljumah A | Clinical Presentation, Risk Factors, and Treatment Modalities of Hepatocellular Carcinoma: A Single Tertiary Care Center Experience | Non-comparative study |
| Bhatti A | Clinical Profile and Treatment of Hepatocellular Carcinoma: A Single-Center Experience | Combined treatments |
| Campigotto M | Comparison between hepatocellular carcinoma prognostic scores: A 10-year single-center experience and brief review of the current literature | Study with missing data |
| Chen YS | Surgical resection significantly promotes the overall survival of patients with hepatocellular carcinoma: a propensity score matching analysis | Solitary lesion classified as stage B |
| Colombo GL | Patterns of treatment and costs of intermediate and advanced hepatocellular carcinoma management in four Italian centers | Non-comparative study |
| Farinati F | BCLC stage B hepatocellular carcinoma and transcatheter arterial chemoembolization: a 20-year survey by the Italian Liver Cancer group | Solitary lesion classified as stage B |
| Guo H | Surgical resection improves long-term survival of patients with hepatocellular carcinoma across different Barcelona Clinic Liver Cancer stages | Solitary lesion classified as stage B |
| Gyedu A | No Patients to Resect or Transplant: An Analysis of Patients with Hepatocellular Carcinoma Admitted to a Major African Referral Hospital | Non-comparative study |
| Hiraoka A | Prediction of Prognosis of Intermediate-Stage HCC Patients: Validation of the Tumor Marker Score in a Nationwide Database in Japan | Non-comparative study |
| Ho MC | Liver Resection Improves the Survival of Patients with Multiple Hepatocellular Carcinomas | Inaccurate BCLC staging |
| Hsu CY | Comparison of Surgical Resection and Transarterial Chemoembolization for Hepatocellular Carcinoma beyond the Milan Criteria: A Propensity Score Analysis | Solitary lesion classified as stage B |
| Jianyong L | Barcelona Clinic Liver Cancer Stage BHepatocellular Carcinoma | Solitary lesion classified as stage B |
| Kariyama K | Treatment of Intermediate-Stage Hepatocellular Carcinoma in Japan: Position of Curative Therapies | Combined treatments |
| Kumar A | Current Practices in Management of Hepatocellular Carcinoma in India: Results of an Online Survey | Non-comparative study |
| Kwak HW | Clinical outcomes of a cohort series of patients with hepatocellular carcinoma in a hepatitis B virus-endemic area | Combined treatments |
| Labgaa I | Surgical Resection Versus Transarterial Chemoembolization for Intermediate Stage Hepatocellular Carcinoma (BCLC-B): An Unsolved Question | Non-comparative study |
| Lacin S | The Effects of Different Treatment Modalities on the Disease Course and Survival in Patients with Hepatocellular Cancer | Non-comparative study |
| Ladron de Guevara L | Hepatocellular carcinoma: Epidemiological profile from a cohort of federal employees in Mexico | Non-comparative study |
| Lin CL | Comparison of surgical resection and transarterial chemoembolization for patients with intermediate stage hepatocellular carcinoma | Solitary lesion classified as stage B |
| Lin CT | Comparing Hepatic Resection and Transarterial Chemoembolization for Barcelona Clinic Liver Cancer (BCLC) Stage B Hepatocellular Carcinoma: Change for Treatment of Choice? | Solitary lesion classified as stage B |
| Longo L | BCLC-B Subclassification and the Hong Kong Liver Cancer System in Intermediate Hepatocellular Carcinoma Identifying Candidates for Curative Therapy | Non-comparative study |
| Mansoor H | Clinical features and survival of patients with hepatocellular carcinoma at a cancer treatment facility | Study with missing data |
| Skladany L | Hepatocellular carcinoma in central Slovakia - tertiary referral centre experience with 207 patients | Inaccurate BCLC staging |
| Suh SW | Predictors of Micrometastases in Patients with Barcelona Clinic Liver Cancer Classification B Hepatocellular Carcinoma | Study with missing data |
| Wang JH | The efficacy of treatment schedules according to Barcelona Clinic Liver Cancer staging for hepatocellular carcinoma – Survival analysis of 3892 patients | Solitary lesion classified as stage B |
| Weinmann A | Survival analysis of proposed BCLC-B subgroups in hepatocellular carcinoma patients | Combined treatments |
| Weinmann A | Trends in Epidemiology, Treatment, and Survival of Hepatocellular Carcinoma Patients Between 1998 and 2009 An Analysis of 1066 Cases of a German HCC Registry | Non-comparative study |
| Xiang X | Distribution of tumor stage and initial treatment modality in patients with primary hepatocellular carcinoma | Study with missing data |
| Xie ZR | Health-Related Quality of Life of Patients with Intermediate Hepatocellular Carcinoma after Liver Resection or Transcatheter Arterial Chemoembolization | Study with missing data |
| Zhao YN | Hepatic resection versus transarterial chemoembolization for patients with Barcelona Clinic Liver Cancer intermediate stage Child-Pugh A hepatocellular carcinoma | Inaccurate BCLC staging |
| Zhong C | A randomized controlled trial of hepatectomy vesus transcatheter arterial chemoembolization for resectable BCLC Stage B/C hepatocellular carcinoma | Inaccurate BCLC staging |
| Zhong JH | Comparison of Long-Term Survival of Patients with BCLC Stage B Hepatocellular Carcinoma after Liver Resection or Transarterial Chemoembolization | Inaccurate BCLC staging |
