# Supplementary figures and images for: Liver resection versus transarterial chemoembolisation for the treatment of intermediate hepatocellular carcinoma: a systematic review and meta-analysis
Source: Int J Surg. 2023 Apr 14;109(5):1439–46. doi: 10.1097/JS9.0000000000000344 (PMC10389385; doi:10.1097/JS9.0000000000000344)

Supplemental Figure S2. Funnel Plot diagram – survival outcome before matching

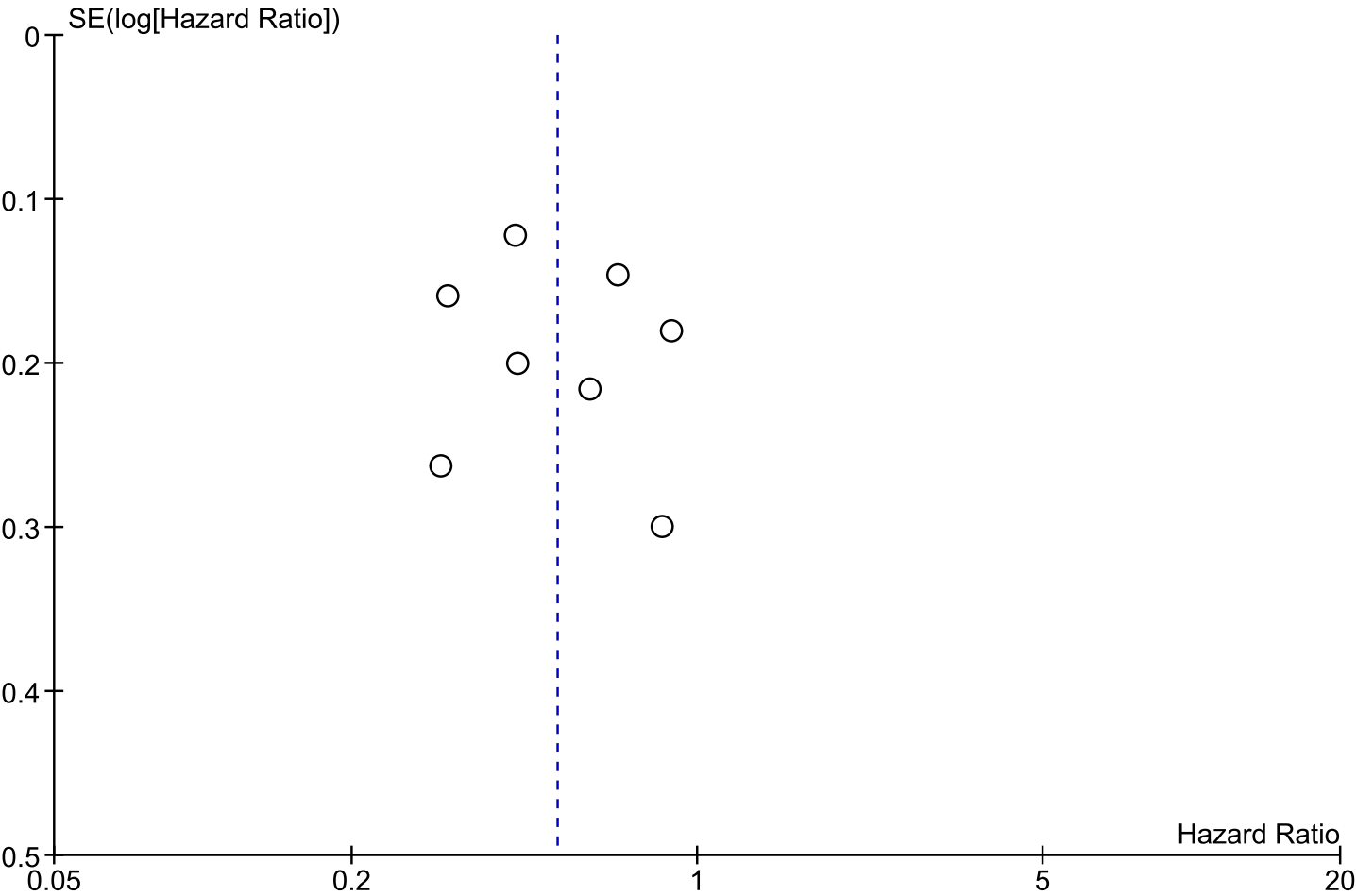

Supplement: Supplementary file 5 [file js9-109-1439-s005.pdf]

Supplemental Figure S3. Funnel Plot diagram – survival outcome after matching

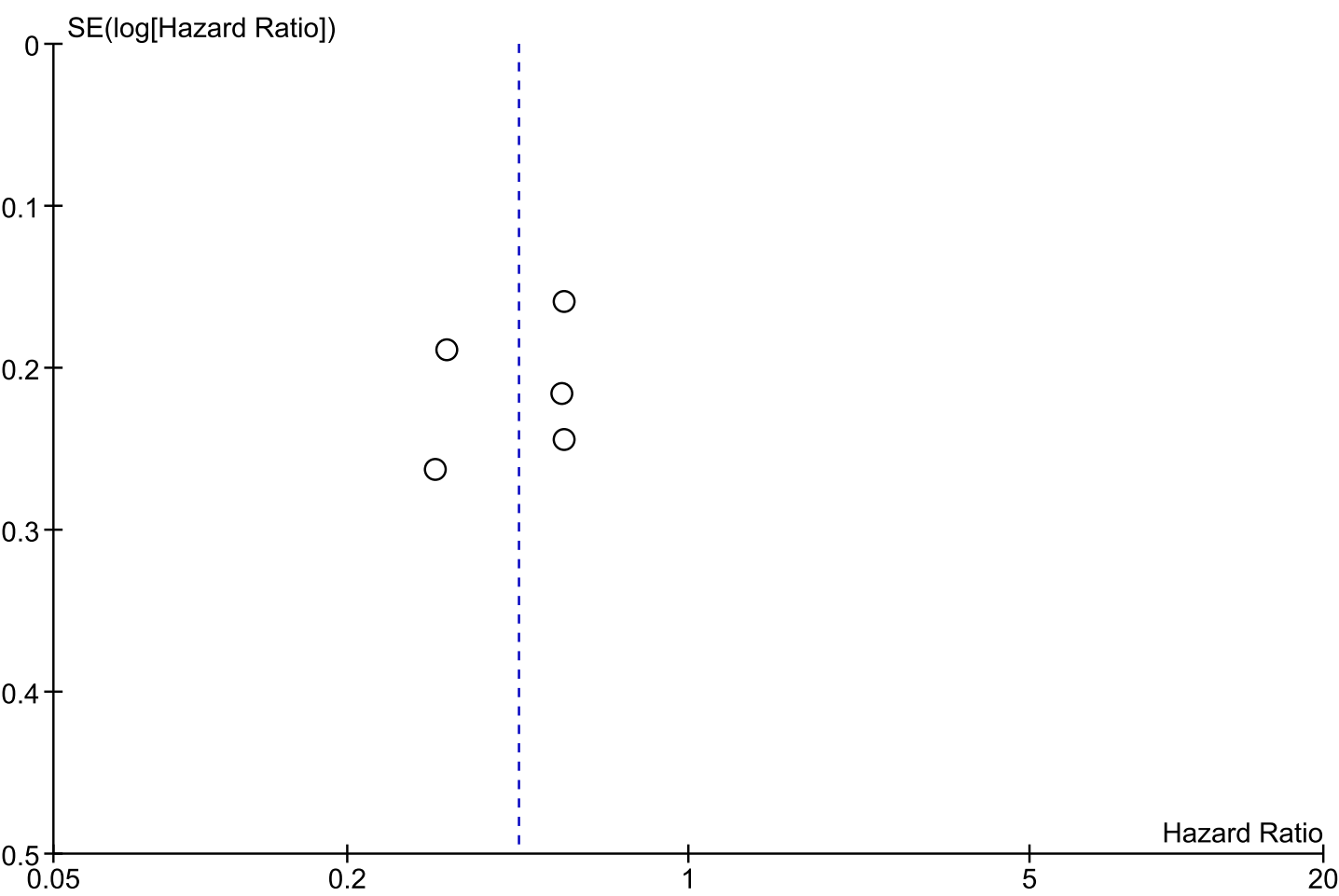

Supplement: Supplementary file 8 [file js9-109-1439-s008.pdf]

Supplemental Figure S1. Sensitivity analysis

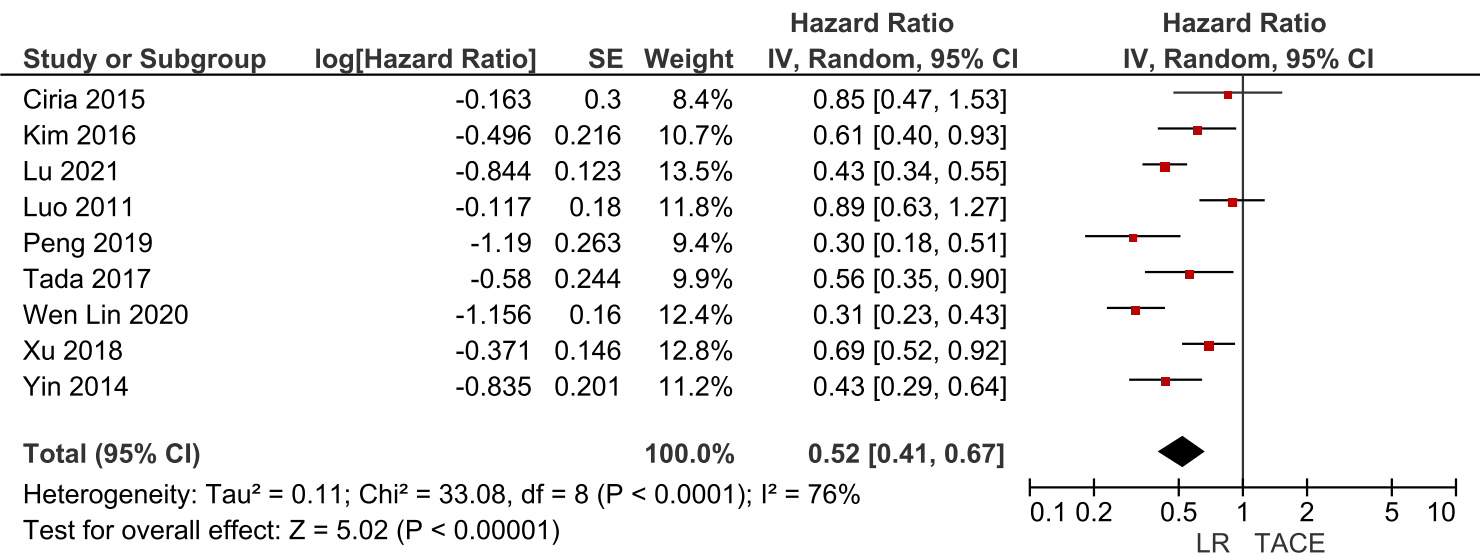

Supplement: Supplementary file 9 [file js9-109-1439-s009.pdf]
