# Supplementary material for: Liver resection versus transarterial chemoembolisation for the treatment of intermediate hepatocellular carcinoma: a systematic review and meta-analysis
Source: Int J Surg. 2023 Apr 14;109(5):1439–46. doi: 10.1097/JS9.0000000000000344 (PMC10389385; doi:10.1097/JS9.0000000000000344)
Supplement: Supplementary file 6 [file js9-109-1439-s006.docx]

Supplemental Table S3. Baseline patients’ clinical characteristics after propensity matching

| First Author | Age (year) | | Male (%) | | HBV (%) | | HCV (%) | | Tumor number | | Maximal tumor diameter | |
| --- | --- | --- | --- | --- | --- | --- | --- | --- | --- | --- | --- | --- |
|  | LR | TACE | LR | TACE | LR | TACE | LR | TACE | LR | TACE | LR | TACE |
| Jun Young Kim^13^ | nr | nr | nr | nr | nr | nr | nr | nr | nr | nr | nr | nr |
| Toshifui Tada^24^ | 69 (63–75) | 69 (63–75) | 83.3 | 82.6 | 18.9 | 14.4 | 57.6 | 64.4 | 2 (2–4)^£^ | 3 (2–4)^£^ | 4.6 (3.6–6.2) | 4 (3.2–5.7) |
| Chih-Wen Lin^25^ | 62 (35–82) | 64 (36–87) | 83.6 | 77 | 50 | 41.4 | 21.4 | 29.9 | 35^¥^ | 47.1^¥^ | 8.2±3.3^χ^ | 8.2±3.5^χ^ |
| Linbin Lu^21^ | 51.6±12.2^χ^ | 52.1±12.7^χ^ | 90.5 | 95.3 | 98.7 | 97.3 | nr | nr | 42 | 40.8 | 6.4±2.7^χ^ | 6.4±3.3^χ^ |
| Yufu Peng^23^ | 54 (31–76) | 57 (29–79) | 86 | 87 | 76 | 79 | nr | nr | 24 | 26 | 5 (3–15.5) | 5 (2–15) |

Values of age and maximal tumor diameter are expressed as median (range) unless indicated otherwise; χ, mean±standard deviation; β, percentage of patients with maximal tumor diameter ≥5cm;

Values of tumor number are expressed as percentage of patients with four or more tumors unless indicated otherwise; £, median (interquartile range); ¥, percentage of patients with three or more tumors

HBV, hepatitis B virus; HCV, hepatitis C virus; LR, liver resection; TACE, transarterial chemoembolisation; nr, not reported
