# Supplementary material for: Liver resection versus transarterial chemoembolisation for the treatment of intermediate hepatocellular carcinoma: a systematic review and meta-analysis
Source: Int J Surg. 2023 Apr 14;109(5):1439–46. doi: 10.1097/JS9.0000000000000344 (PMC10389385; doi:10.1097/JS9.0000000000000344)
Supplement: Supplementary file 7 [file js9-109-1439-s007.docx]

Supplemental Table S4. Survival outcome after propensity matching

| First Author | Median Follow-up (months) | | 1-Year Survival (%) | | 3-Year Survival (%) | | 5-Year Survival (%) | | p |
| --- | --- | --- | --- | --- | --- | --- | --- | --- | --- |
|  | LR | TACE | LR | TACE | LR | TACE | LR | TACE |  |
| *Jun Young Kim^13^ | nr | nr | nr | nr | nr | nr | nr | nr | <0.001 |
| Linbin Lu^21^ | nr | nr | nr | nr | nr | nr | nr | nr | <0.0003 |
| Yufu Peng^23^ | nr | nr | 83 | 75 | 56 | 15 | nr | nr | <0.001 |
| Toshifumi Tada^24^ | 30 | 28 | nr | nr | 63.4 | 53.0 | 53.1 | 34.1 | 0.02 |
| Chih-Wen Lin^25^ | nr | nr | 89.2 | 70.1 | 69.4 | 36.3 | 61.2 | 15.7 | <0.0001 |

LR, liver resection; TACE, transarterial chemoembolisation; nr, not reported

* Study reported Hazard Ratio related to treatment modality after a matched analysis
